# 2122. The Impact of HHV-6 DNAemia on hematopoietic cell transplant (HCT) recipients at high risk for CMV reactivation in the era of Letermovir

**DOI:** 10.1093/ofid/ofac492.1743

**Published:** 2022-12-15

**Authors:** Krithia Srinivasan, Amy Spallone, Fareed Khawaja, Joseph Sassine, Oscar Morado Aramburo, Anthony J Febres-Aldana, Gabriella Rondon, Jeremy Ramdial, Elizabeth Shpall, Ella Ariza-Heredia, Roy F Chemaly

**Affiliations:** Stanford, Palo Alto, California; University of Texas MD Anderson Cancer Center, Houston, Texas; The University of Texas MD Anderson Cancer Center, Houston, Texas; University of Oklahoma Health Sciences Center, Oklahoma City, Oklahoma; The University of Texas MD Anderson Cancer Center, Houston, Texas; The University of Texas MD Anderson Cancer Center, Houston, Texas; The University of Texas MD Anderson Cancer Center, Houston, Texas; University of Texas MD Anderson Cancer Center, Houston, Texas; The University of Texas MD Anderson Cancer Center, Houston, Texas; The University of Texas MD Anderson Cancer Center, Houston, Texas; MD Anderson, Houston, Texas

## Abstract

**Background:**

Letermovir (LTV) has reduced non-relapse mortality (NRM) in allogeneic hematopoietic cell transplant (allo-HCT) recipients by reducing the rate of clinically significant cytomegalovirus infections (CS-CMVi). The impact of LTV prophylaxis (PP) on other infections is unclear. We investigated the effects of LTV on human herpes virus 6 (HHV6) DNAemia in HCT recipients with or without CS-CMVi and studied the interaction of HHV6 DNAemia with CS-CMVi and its impact on NRM.

**Methods:**

We performed a single center, retrospective cohort study from March 2016 to December 2018 of consecutive allo-HCT recipients who are CMV recipient seropositive (R+) with or without LTV prophylaxis. Baseline characteristics and infectious complications data were collected. Outcomes of interest was NRM at 100 days, 24 weeks and 48 weeks post allo-HCT. Univariate analysis was performed to identify risk factors for HHV6 reactivation within the first year including CS-CMVi and risk factors for NRM at 48 weeks post transplant. A logistic regression was performed to identify independent risk factors for HHV6 DNAemia and NRM at 48 weeks. Patients with relapse were excluded from NRM analysis.

**Results:**

A total of 539 allo-HCT recipients were included in our analysis; 124 (23%) with and 415 (77%) without LTV PP. HHV6 DNAemia was identified in 111 (21%) allo-HCT recipients within the first year of transplant, where CS-CMVi occurred in 241 (45%) (table 1). Risk factors for HHV6 DNAemia included African American race, underlying ALL, Haploidentical or cord HCT, marrow or cord source of stem cells, use of Post-cyclophosphamide, and CS-CMVi. On multivariate analysis, CS-CMVi was the only independent predictor of HHV6 reactivation (Adjusted OR: 1.69) (table 1). Independent predictors of NRM on logistic regression included CS-CMVi (OR: 1.67, CI 95% 1.03-2.62), and age > 40 years (OR: 2.21, CI 95% 1.24-3.95), and matched related donor allo-HCT (OR: 0.36, CI 95% 0.18-0.70) as a protective factor (table 2).

**Figure:**
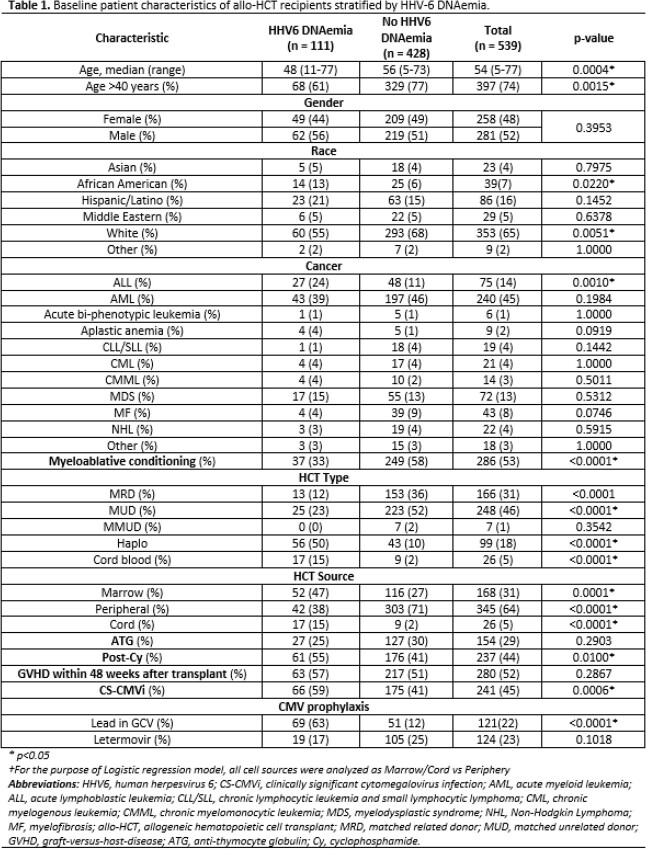

**Figure:**
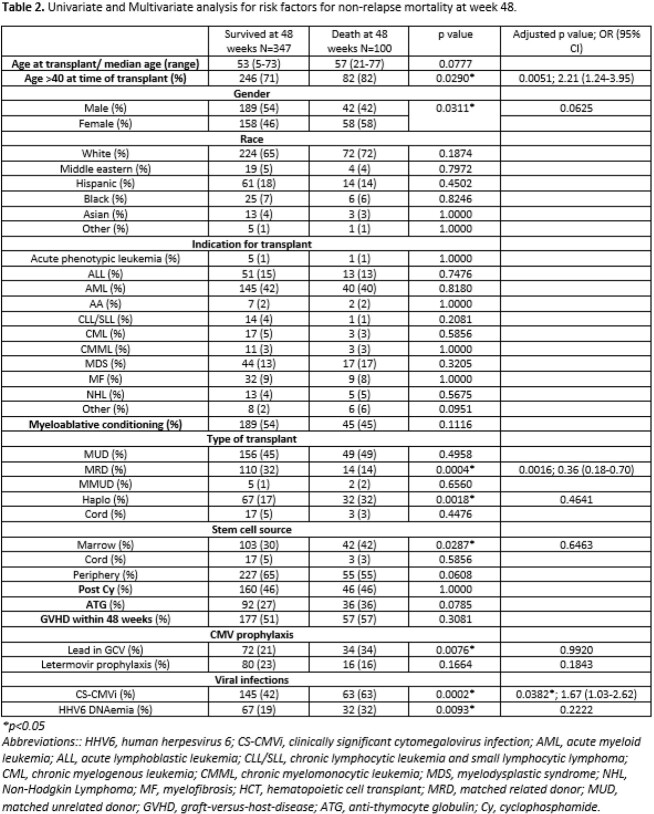

**Conclusion:**

HHV6 DNAemia is strongly associated with CS-CMVi, which is probably a reflection of poor T cell recovery post HCT such as Haploidentical or Cord blood HCT. Furthermore, CS-CMVi was associated with NRM whereas HHV6 DNAemia was not. Larger studies are needed to better elucidate this interaction.

**Disclosures:**

**Gabriella Rondon, MD**, Omeros: Advisor/Consultant **Elizabeth Shpall, MD**, Adaptimmune: Advisor/Consultant|Affimed: License agreement|Axio: Advisor/Consultant|Bayer Helathcare Pharmaceuticals: Honoraria|Fibroblasts and FibrioBiologics: Advisor/Consultant|Navan: Advisor/Consultant|NY Blood Center: Advisor/Consultant|Takeda: License agreement **Ella Ariza-Heredia, MD**, MERCK: Grant/Research Support **Roy F. Chemaly, MD/MPH**, Karius: Advisor/Consultant|Karius: Grant/Research Support.

